# Supplementary material for: Therapeutic Targeting of Fumaryl Acetoacetate Hydrolase in Hereditary Tyrosinemia Type I
Source: Int J Mol Sci. 2021 Feb 11;22(4):1789. doi: 10.3390/ijms22041789 (PMC7916972; doi:10.3390/ijms22041789)
Supplement: Supplementary file 1 [file ijms-22-01789-s001.pdf]

Supplementary Material for:

Therapeutic targeting of fumaryl acetoacetate hydrolase in hereditary tyrosinemia type I

Jon Gil-Martínez[1,#], Iratxe Macías[1,#], Luca Unione[1,2], Ganeko Bernardo-Seisdedos[2], Fernando Lopitz-Otsoa[1], David Fernandez-Ramos[1], Ana Laín[1], Arantza Sanz-Parra[1], José M Mato[1] and Oscar Millet[1,2,*]

[#]Both authors equally contributed to the work.

[1]Precision Medicine and Metabolism Laboratory, CIC bioGUNE, Bizkaia Technology Park, Bld. 800, 48160 Derio, Bizkaia, Spain.

[2]ATLAS Molecular Pharma, Bizkaia Technology Park, Bld. 800, 48160 Derio, Bizkaia, Spain.

*To whom correspondence should be addressed: Oscar Millet: Protein Stability and Inherited Disease Laboratory, CIC bioGUNE, Bizkaia Technology Park, Bld. 800, 48160 Derio, Bizkaia, Spain. Tel: (+34) 946 572 504; Fax: +34 946 572 502; **omillet@cicbiogune.es**

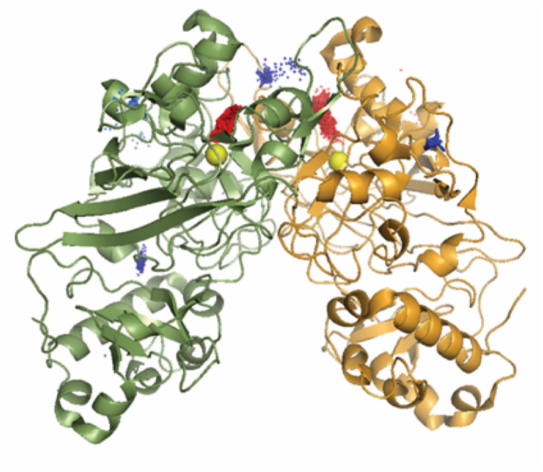

Figure S1. Druggable binding sites. Docking hits of a virtual screening of 20.000 compounds located in the dimeric structure of human FAH. Hits in and out of the active site are colored in red and blue respectively.

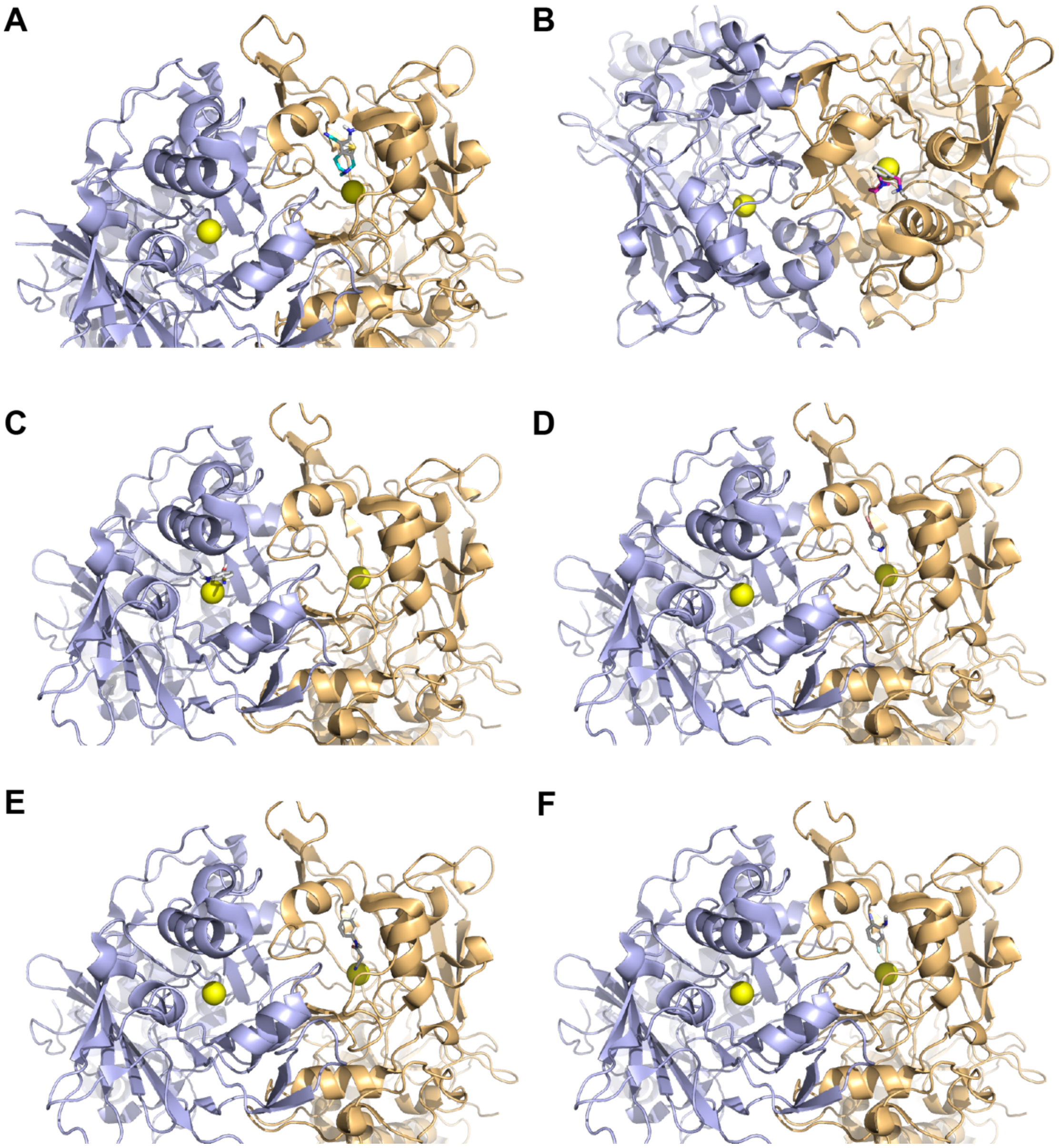

Figure S2. Docking models for the set of compounds. A) 3E.11. B) 6A.11. C) 8H.11. D) 30B.10. E) 23A.11. F) 27H.4

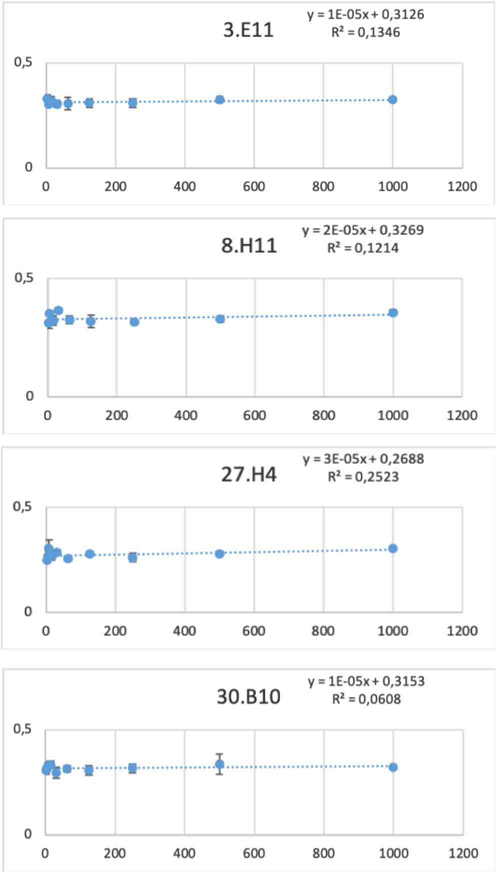

Figure S3. Cell viability assays and IC_{50} determination. Cell viability assay for four representative chemicals determined in M1 cell lines, as indicated. Absorbance at 550 nm monitors the tetrazolium reaction in the mitochondria and is a reporter of cell viability.
